# Perinatal listeriosis patients treated at a maternity hospital in Beijing, China, from 2013–2018

**DOI:** 10.1186/s12879-020-05327-6

**Published:** 2020-08-14

**Authors:** Chunyun Li, Huihui Zeng, Xin Ding, Yi Chen, Xiaowei Liu, Li Zhou, Xin Wang, Yumei Cheng, Shanshan Hu, Zheng Cao, Ruixia Liu, Chenghong Yin

**Affiliations:** 1 Department of Internal Medicine, Capital Medical University Beijing Obstetrics and Gynecology Hospital, No. 251 Yaojiayuan Road, Chaoyang District, Beijing, 100026 P. R. China; 2 Department of Neonatology, Capital Medical University, Beijing, China; 3 Department of Obstetrics, Capital Medical University, Beijing, China; 4 Department of Disease Prevention and Control and Nosocomial Infection, Capital Medical University, Beijing, China; 5 Department of Clinical Laboratory, Capital Medical University, Beijing, China; 6 Department of Central Laboratory, Capital Medical University Beijing Obstetrics and Gynecology Hospital, 251 Yaojiayuan Road, Chaoyang District, Beijing, 100026 P. R. China

**Keywords:** *Listeria monocytogenes*, Listeriosis, Foodborne infectious disease, Perinatal, Maternal, Neonate, Septicemia, Central nervous system infection

## Abstract

**Background:**

Listeriosis is a rare but severe foodborne infectious disease. Perinatal listeriosis is often associated with septicemia, central nervous system (CNS) infection, and serious adverse pregnancy outcomes (miscarriage and neonate death). Here we report the characteristics and outcomes of perinatal listeriosis cases treated over 6 years at Beijing Obstetrics and Gynecology Hospital (BOGH), the largest maternity hospital in China.

**Methods:**

We retrospectively reviewed the records of laboratory-confirmed, pregnancy-associated listeriosis cases treated from January 1, 2013 to December 31, 2018. The clinical manifestations, laboratory results, perinatal complications and outcomes (post-natal follow-up of 6 months) were investigated.

**Results:**

In BOGH, 12 perinatal listeriosis cases were diagnosed based on *Listeria monocytogenes* positive culture, including 10 single pregnancies and 2 twin pregnancies. The corresponding incidence of pregnancy-associated listeriosis was 13.7/100,000 deliveries. Among those cases, four pregnant women and four newborns had septicemia, and two of the neonates with septicemia also suffered CNS infection. All the maternal patients recovered. Two inevitable miscarriages and four fetal stillbirths occurred. Of the eight delivered newborns, six survived, and two died within 2 days from birth. None of the survivors had neurological sequelae during a 6-month follow-up. The overall feto-neonatal fatality rate was 57.1%; notably, this rate was 100% for infections occurring during the second trimester of pregnancy and only 14.3% for those occurring in the third trimester.

**Conclusions:**

Perinatal listeriosis is associated with high feto-neonatal mortality, and thus, a public health concern. Additional large-scale studies are needed to strengthen the epidemiological understanding of listeriosis in China.

## Background

*Listeria monocytogenes* (*L. monocytogenes*) is a facultative anaerobic Gram-positive bacterium that causes severe foodborne illnesses associated with substantial mortality (20–30%) [1]. This pathogen, a ubiquitous bacterium in nature, can be isolated from soil, stream water, vegetables, fruits, raw meat, milk products, ready-to-eat food products, and even refrigerated processed foods because Listeria can survive and grow at wide ranges of pH and temperatures as well as high salt concentrations [2–4].

*L. monocytogenes* is mainly transmitted through the consumption of contaminated foods. After crossing the intestinal mucosal barrier, *L. monocytogenes* disseminate within the circulation and show preferential accumulation in the central nervous system (CNS) and the placenta [5]. Increased progesterone levels during pregnancy weaken the cellular immunity, which makes expectant mothers particularly susceptible to microorganisms such as like *L. monocytogenes* [6, 7]. The infection risk for pregnant women is 12–20 times higher than that for the general population [8, 9]. The reported incidence of pregnancy-related listeriosis has ranged 4.3–25 cases per 100,000 births [10–13]. In systematic reviews, pregnancy-associated cases have accounted for 20.7–43% of all listeriosis cases worldwide [14, 15]. In China, a human listeriosis surveillance system was established in 2013. However, to date, listeriosishas not become a notifiable disease in China. Two recent reports showed that in China, perinatal listeriosis accounts for 41.1–52% of clinical listeriosis cases, and thus, the burden of pregnancy-related listeriosis in the country is not light [16, 17]. Pregnant women infected with *L. monocytogenes* are often asymptomatic or have only nonspecific clinical symptoms such as gastrointestinal and flu-like symptoms. However, many of these patients experience adverse pregnancy outcomes, including fetal loss, preterm birth, and neonatal listeriosis.

This report retrospectively reviewed all laboratory-confirmed, pregnancy-associated listeriosis cases treated from January 2013 to December 2018 at Beijing Obstetrics and Gynecology Hospital (BOGH), a high-level maternal and child health care hospital with 660 beds in Beijing, China.^1^ In the present study, we detail the clinical characteristics and outcomes of these *L. monocytogenes* infected perinatal patients.

## Methods

The number of births in BOGH exceeds 14,000 every year. We retrospectively analyze the clinical data of all laboratory-confirmed, pregnancy-associated *L. monocytogenes* infections treated from January 1, 2013 to December 31, 2018, based on a list generated from the Department of Disease Prevention and Control and Nosocomial Infection of BOGH. Recording of the clinical information of all patients of BOGH in an electronic database began in 2013.

Pregnancy-associated listeriosis cases includes illness with an onset during pregnancy or within the first 2 weeks of the postpartum period as well as illness in the neonate between birth and 4 weeks of age [1]. All the confirmed cases were based on the isolation of *L. monocytogenes* from a normally sterile site (e.g., blood or cerebrospinal fluid [CSF]) or products of conception (e.g., placental or fetal tissue), with the presence of compatible clinical symptoms. A perinatal listeriosis case was defined based on isolation of *L. monocytogenes* from a clinical sterile sample from the pregnant woman or foetus, stillborn, and newborn aged < 4 weeks [9]. If *L. monocytogenes* was isolated in samples from both the mother and neonate, a single case was counted. Neonatal cases were divided into early onset (diagnosed between birth and day 6) and late onset (diagnosed between 7 and 28 days) [1, 18]. *L. monocytogenes* CNS infection was diagnosed if *L. monocytogenes* was isolated from a patient’s CSF or when a patient had neurological symptoms (e.g., altered consciousness, seizures, nuchal rigidity, or focal neurological symptoms, and an increased white blood cell [WBC] count in the CSF) and blood culture showing *L. monocytogenes*. If a patient did not meet the criteria for CNS infection diagnosis but *L. monocytogenes* was found on blood culture, the patient was considered to have septicemia [19].

The culture, isolation, and identification of *L. monocytogenes* was performed using the traditional blood agar plating method followed by automated biochemical confirmation (bioMérieux VITEK 2 COMPACT, France) with visible colonies [19].

We defined ‘stillbirth’ as death of the fetus between 24 and 41 weeks of gestation, and fetal loss before 24 weeks was defined as an inevitable miscarriage. Furthermore, we calculated the overall neonatal fatality of pregnancy-associated listeriosis, including miscarriages, stillbirths and newborn deaths.

We used descriptive statistics in this study. Where appropriate, data are expressed as mean ± standard deviation (SD).

## Results

### Basic characteristics of perinatal listeriosis cases in the considered period

**Fig. 1:**
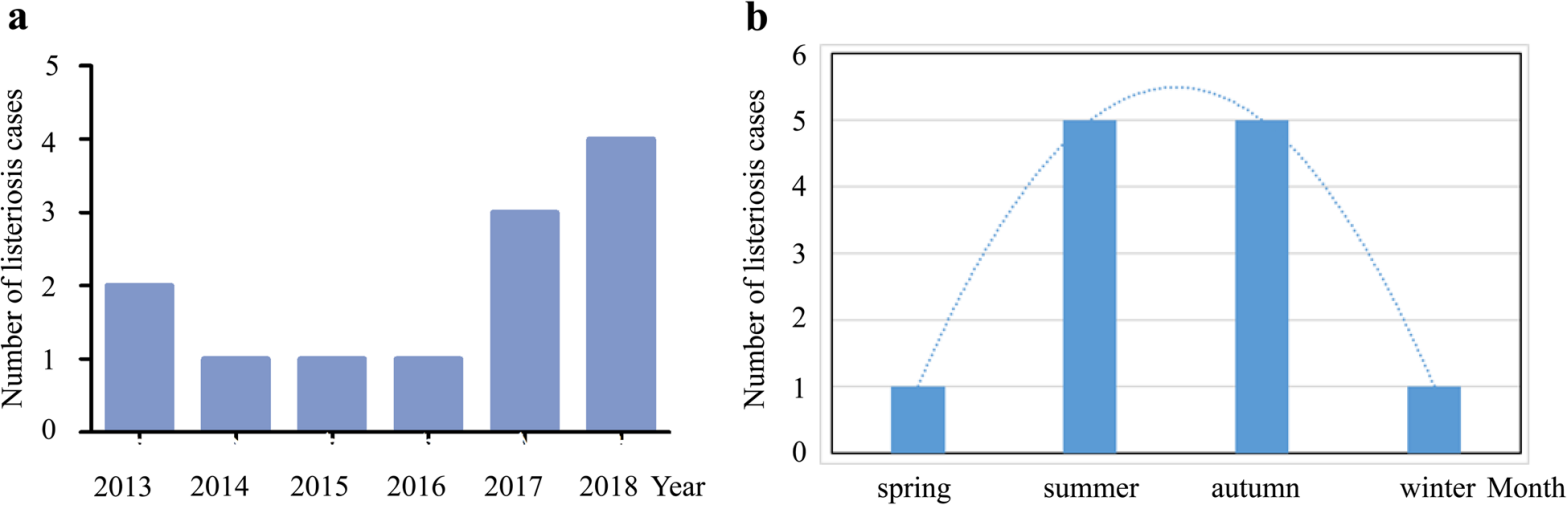
The annual and seasonal numbers of pregnancy-associated listeriosis cases from 2013 to 2018. **a** Annual number of perinatal listeriosis infections. **b** Seasonal number of perinatal listeriosis cases. Spring (March, April, May), summer (June, July, August), fall (September, October, November), and winter (December, January and February)

We identified 12 cases of pregnancy-associated listeriosis from overall total of 87,644 deliveries, for an incidence of 13.7/100,000 births. These included 12 maternal and 14 neonatal infections with *L. monocytogenes*, and the characteristics of these cases are summarized in Table 1. The annual and seasonal numbers of pregnancy-associated listeriosis cases are shown in Fig. 1a and b, respectively. Ten cases (10/12 ≈ 83.33%) occurred during summer and fall months (i.e. June to November) during 2013 to 2018. The median age of these women was 29 years (range, 25–41 years).

**Table 1:** Characteristics of 12 maternal and 14 neonatal cases of pregnancy-associated listeriosis

Group	Maternal	Neonatal
Total, n	12	14
Median age (min, max), y	29 (25, 41)	
Median gestation at delivery (min, max), wk		29.0 (20.0, 38.1)
Underlying disease, n (%)		
GDM	2 (16.67)	
SLE	1 (8.33)	
Clinical manifestations, n (%)		
Fever	11 (91.67)	
Gastrointestinal symptoms	5 (41.67)	
Flu-like symptoms	3 (25)	
Laboratory findings		
Peripheral WBC, mean ± SD, 10^9^/L	21.13 ± 6.55	
Neutrophils, %, median (min, max)	82.55 (74.3, 92.6)	
Mononuclear cells, mean ± SD, 10^9^/L	0.98 ± 0.34	
Mortality, n (%)	0	8 (57.1)

Abbreviations: max, maximum; min, minimum; *GDM* gestational diabetes mellitus, *SLE* systemic lupus erythematosus, *WBC* white blood cell, *SD* standard deviation

### Clinical characteristics of maternal listeriosis cases

The clinical characteristics of the 12 maternal listeriosis cases are described in Table 2. Among them, 10 were singleton pregnancies and 2 were twin pregnancies (i.e., cases 2 and 9). Six cases were infected with *L. monocytogenes* in the second trimester pregnancy (between 14 and 27 weeks), and the other six were infected in the third trimester pregnancy (between 28 and 41 weeks). Their median gestational age at the time of infection was 29.3 weeks (range, 20.0–38.1 weeks). Eleven of the twelve maternal listeriosis patients had prenatal fever (38–39.3 °C), and 3 of 12 had flu-like symptoms. Five patients had gastrointestinal symptoms (diarrhea and abdominal pain). Various obstetrical symptoms were reported among the 12 pregnant women, including decreased fetal movement (*n* = 5), intrauterine fetal death (*n* = 2), premature rupture of the membranes (PROM, *n* = 2), and vaginal bleeding (*n* = 1). All of the symptomatic women received antibiotic therapy, and eight patients received only cephalosporin antibiotics initially.

**Table 2:** Clinical characteristics of 12 maternal listeriosis cases

No	Gestation (wk)	Obstetrical manifestations	Culture sites				Initial antibiotic	Switched antibiotic	Maternal complications and outcomes
			Maternal blood	Placental tissue	Cervical secretion	Others			
1	31.3	decreased fetal movement 3d; fetal distress; Tmax:39.3 °C	(−)	(+)	/	/	metronidazole + amoxicillin 4d	No	premature delivery; C-section; severe meconium staining of amniotic fluid; recovered
2	24.4	decreased fetal movement 3d; lower abdominal pain; twin pregnancy; PROM; Tmax:38.5 °C	(−)	(+)	(+)	/	cefuroxime 3d	No	stillbirth; recovered
3	20.0	abdominal pain; fetal movement disappear 3d; chill; Tmax:38.7 °C	(−)	(+)	(−)	hydrothorax, ascite, amniotic fluid (+)	amoxicillin 3d	No	inevitable miscarriage; induced labor; recovered
4	34.3	decreased fetal movement 1d; uterine contraction; fetal distress; headache; Tmax:38.8 °C	(−)	(+)	(−)	/	cefmetazole 1d	moxifloxacin 6d	premature delivery; severe meconium staining of amniotic fluid; recovered
5	26.0	lower abdominal pain 17 h; PROM; fetal distress; septicemia; Tmax:39 °C	(+)	(+)	(−)	/	cefuroxime 3d	metronidazole 3d	stillbirth; induced labor; recovered
6	36.1	decreased fetal movement 2d; fetal distress; polyhydramnios; Tmax:38 °C	(−)	(+)	/	/	cefuroxime 3d	No	premature delivery; C-section; severe meconium staining of amniotic fluid; recovered
7	38.1	fetal distress; Tmax:39.2 °C	(−)	(+)	/	/	metronidazole + ceftriaxone 4d	No	normal labor; postpartum hemorrhage; C-section; severe meconium staining of amniotic fluid; recovered
8	21.7	fever15d; intrauterine fetal death1d; lower abdominal pain; headache; Tmax:39.1 °C	(−)	(+)	(−)	/	cefuroxime 8d + cefdinir 5d	moxifloxacin 6d	inevitable miscarriage; induced labor; recovered
9	35.6	fetal distress; threatened prematurity; twin pregnancy; septicemia; Tmax:38.4 °C	(+)	(−)	/	/	ceftriaxone 5d	No	premature delivery; postpartum hemorrhage; recovered
10	24.9	fever7d; fetal distress; abdominal pain; septicemia; Tmax:39.3 °C	(+)	(+)	(−)	/	ceftriaxone 7d	PNG 4d	stillbirth; recovered
11	35.4	fetal distress; decreased fetal movement 1d; afebrile	(−)	(+)	/	/	cefuroxime 3d	No	premature delivery; recovered
12	27.3	fever 2w; prematurity; vaginal bleeding; uterine contraction; septicemia; Tmax:39 °C	(+)	(+)	(−)	/	clindamycin + ceftriaxone 2d	azithromycin + PNG 7d	premature delivery; postpartum hemorrhage; C-section; mild meconium staining of amniotic fluid; recovered

Abbreviations: *Tmax* maximal temperature, *PROM* premature rupture of the membranes, *C-section* cesarean section, *PNG* penicillin-G

Among the 12 maternal patients, 1 progressed to normal labor, 5 experienced fetal loss and 6 had premature deliveries. Additional complications included postpartum hemorrhage in 3 patients, meconium staining in 5 patients, and the need for induced labor in 3 patients. Caesarean section (C-section) was performed in four cases, due to abnormal fetal heart rates. None of the women were diagnosed with CNS infection. One-third (4/12) had septicemia. All of the maternal patients eventually recovered after delivery with no sequelae.

### Clinical characteristics and outcomes of listeriosis infections in offspring

The clinical characteristics and outcomes of all 14 offspring are summarized in Table 3. Nine of the fourteen offspring (64.29%) were female, and the median birth weight was 1305 g (range, 380–3565 g). Six of the fourteen neonates were found to have confirmed *L. monocytogenes* infection based on microbiological methods (laryngeal swab: 2; blood + laryngeal swab: 2; blood + CSF + laryngeal swab: 2). Two neonates were diagnosed with CNS infection, and four had septicemia. No late-onset cases of newborn/infant listeriosis were observed. Two inevitable miscarriages and four fetal stillbirths occurred, and the other eight neonates presented with fetal distress. Four newborns received intubation. Of the eight delivered newborns, six survived, and two died within 2 days after birth. None of the survivors had neurological sequelae throughout a 6-month follow-up.

**Table 3:** Clinical characteristics and outcomes among the 14 neonates in the 12 pregnancy-associated listeriosis cases

No.	Birth weight (g)	Presentation	Culture sites			Initial antibiotic	Switched antibiotic	Intubation	Complications	Outcomes
			Feto-neonatal blood	CSF	Laryngeal swab					
1	1610	fetal distress; Apgar 6; SpO_2_ 92% on ambient air; rash	(−)	/	(+)	NA	NA	Yes	preterm birth; neonatal asphyxia; pneumonia; hypoglycemia, DIC; septic shock; renal failure; hyperlactacidemia; metabolic acidosis	deceased day 2; infant listeriosis
2.1 & 2.2	740/560	fetal distress; decreased fetal movement	/	/	/	/	/	/	stillbirth; induced labor	death
3	440	fetal distress; decreased fetal movement	/	/	(+)	/	/	/	inevitable miscarriage; induced labor	infant listeriosis
4	2275	fetal distress; Apgar 9; SOB; SpO_2_ 80% on ambient air; rash	(+)	(+)	(+)	NA	NA	Yes	septicemia; preterm birth; CNS infection; meningitis; intrauterine infection; low birth weight; hypoglycemia; DIC; sepsis; thrombocytopenia; myoardial infarction	survived; infant listeriosis
5	1000	fetal distress	/	/	/	/	/	/	stillbirth	death
6	2565	fetal distress; Apgar 10; SpO_2_ 95% on ambient air	(+)	/	(+)	cefotoxime + PNG 5d	meropenem + PNG 9d	No	septicemia; preterm birth; intrauterine infection	survived; infant listeriosis
7	3565	fetal distress; Apgar 8; SpO_2_ 88% on ambient air; meconium aspiration	(+)	/	(+)	piperacillin 3d	ceftazidime + vancomycin 12d	Yes	septicemia; intrauterine infection; pneumonia; hyperlactacidemia; neonatal encephalopathy; anemia; myoardial infarction; thrombocytopenia	survived; infant listeriosis
8	380	intrauterine fetal death	/	/	/	/	/	/	inevitable miscarriage	induced labor
9.1	2730	fetal distress; Apgar 8; SOB; SpO_2_ 92% on ambient air	(−)	(−)	(−)	cefepime + PNG 10d	No	No	preterm birth; pneumonia; patent foramen ovale	survived
9.2	2285	fetal distress; Apgar 7; SOB; SpO_2_ 90% on ambient air	(+)	(+)	(+)	meropenem + PNG 19d	No	No	preterm birth; septicemia; low birth weight; CNS infection; meningitis; liver function lesion, pneumonia; metabolic acidosis; hypoglycemia; anemia, neutropenia; patent foramen ovale; patent ductus arteriosus	survived; infant listeriosis
10	620	fetal distress	/	/	/	/	/	/	stillbirth	death
11	2810	fetal distress; Apgar 10; SpO_2_ 96% on ambient air; cyanosis	(−)	/	(−)	PNG + latamoxef 7d	cefepime 7d	No	preterm birth; pneumonia; neonatal infection; hypocalcemia; neonatal jaundice; patent foramen ovale; hydronephrosis	survived
12	820	fetal distress; Apgar 8; SpO_2_ 94% on ambient air	/	/	(−)	/	/	Yes	extremely low birth weight; preterm birth; hyperlactacidemia; NRDS	deceased day 1

Abbreviations: *NA* not available, *C-section* cesarean section, *SpO*_*2*_ oxygen saturation from pulse oximetry, *SOB* shortness of breath, *DIC* disseminated intravascular coagulation, *NRDS* neonatal respiratory distress syndrome, *CSF* cerebrospinal fluid, *PNG* penicillin

## Discussion

The incidence of perinatal listeriosis in BOGH was 13.7/100,000 deliveries, which was consistent with previously reported incidence rates [10–13]. In our study, half of the pregnancy-associated listeriosis cases (6/12, cases 2, 3, 5, 8, 10, and 12) suffered from listeriosis in the second trimester of pregnancy, and all seven offspring of these patients died. Among the other six perinatal cases (cases 1, 4, 6, 7, 9, and 11; 7 newborns in total), six newborns survived without sequelae, despite CNS infection in two infants and septicemia in four infants, while one (case 1, delivered at week 31.3) died 2 days after birth. Consistent with the previous studies [13, 20], in this study, the prognosis of neonate from maternal listeriosis cases occurring during late pregnancy was quite excellent even if the infants were infected with *L. monocytogenes*, whereas the pregnancy outcomes of perinatal cases infected prior to the 28th week of gestation were very bad. The overall cases-fatality rate among the offspring was 57.1%, which was close to the range of 32.7–50.7% reported in two recent systematic reviews in the mainland of China [16, 17]. Accordingly, the feto-neonatal listeriosis mortality rate in China is very high, which was in contrast to the low child mortality estimated by de Noordhout et al. [14] in 2010.

We found that 66.67% (8/12) of maternal patients received only cephalosporin antibiotics initially, because cephalosporins are the preferred empirical therapy for obstetric infections with nonspecific clinical symptoms in China. However, while *L. monocytogenes* strains are susceptible to many antibiotics (e.g., ampicillin and penicillin G [PNG]), they are not sensitive to cephalosporins [21–24]. Previous studies demonstrated that delayed diagnosis and inappropriate antibiotic administration decrease the probability of a favorable outcome among *L. monocytogenes* infection cases [20, 25]. In China, there are still no national guidelines for the treatment of listeriosis. According to Hof et al. [22], when listeriosis is a likely diagnosis, the use of ampicillin, PNG, or vancomycin provides empiric coverage for *L. monocytogenes*. However, empiric therapy for bacterial meningitis with ampicillin may not be necessary for children beyond the neonatal period [26]. Ampicillin or PNG, with or without aminoglycoside or gentamicin, is recommended for all forms of listeriosis. Trimethoprim-sulfamethoxazole can be used as an alternative treatment. Two to three weeks of therapy is sufficient for most forms of listeriosis. Rhombencephalitis with abscess formation in the CNS may require 4-week therapy [25, 27]. Notably though, researchers recently found high resistance levels of *L. monocytogenes* to many antibiotics (ampicillin, PNG, tetracycline, cefotaxime, etc.) among clinical and food isolates, which represents a serious problem for the treatment of listeriosis [28, 29].

Listeriosis is a typical foodborne disease, which means food contamination is the major source of infection. Data from Beijing Centers for Disease Control and Prevention (CDC) showed that among the 12 maternal patients in the present study, positive culture of the same strain of *L. monocytogenes* from samples obtained from the patient’s kitchen was observed only for case 5. Even for this case though, various food sources of *L. monocytogenes* made it difficult to trace the pathogen and identify the specific food source.

Inspection reports have shown that the estimated prevalence of *L. monocytogenes* in Chinese food products is 4.42% [30], and in the retail markets of Beijing 15.20% of the raw pork was found to be contaminated [31]. Thus, food safety regarding *L. monocytogenes* contamination remains a problem in China. Additionally, processed food and pasteurized dairy products stored in a refrigerator are also vulnerable to recontamination, because *L. monocytogene* can grow very well at 4 °C [32, 33]. Currently, there are no diet guidelines for the prevention of *L. monocytogenes* infection in China. Therefore, improving awareness of relevant food safety among the people at higher risk of *L. monocytogenes* infection, especially pregnant women and immune-compromised individuals, can facilitate prevention. Because doctors are the most credible source of health information for pregnant patients, campaigns by doctors communicating healthy diet habits education (e.g., avoiding direct eat consumption of even pasteurized dairy products and cooked foods stored in the refrigerator without recooking, and eating out less often) are recommended for pregnant women.

## Conclusions

There are several limitations in this study. First, this was a retrospective study over a protracted timespan of 6 years, and no stored samples were available for further research, such as microbial typing. Second, the lack of epidemiological investigations does not allow for identification of the food sources of *L. monocytogenes* in these cases and the formulation of clear recommendations. Third, the sample size was relatively small. Data large-scale investigation of pregnancy-associated listeriosis is needed to further understanding the demographic distribution of this dangerous infection in China.

## Data Availability

The raw data are not publicly available, but as the medical staff at the hospital, we have access to it. All data generated or analyzed during this study are included in this published article.

## Abbreviations

CNS: Central nervous system
BOGH: Beijing Obstetrics and Gynecology Hospital;
*L. monocytogenes*: *Listeria monocytogenes*
CSF: Cerebrospinal fluid
WBC: White blood cell
PNG: Penicillin-G
SLE: Systemic lupus erythematosus
GDM: Gestational diabetes mellitus
C-section: Caesarean section
PROM: Premature rupture of the membranes
NA: Not available
SpO_2_: Oxygen saturation from pulse oximetry
SOB: Shortness of breath
DIC: Disseminated intravascular coagulation
NRDS: Neonatal respiratory distress syndrome
max: Maximum
min: Minimum
SD: Standard deviation

## Footnotes

1 http://www.bjogh.com.cn/english/article/19730.html
